# Chinese Readers Process Word Class of Parafoveal Words During Sentence Reading

**DOI:** 10.1111/cogs.70197

**Published:** 2026-03-24

**Authors:** Zijun Qi, Yue Xi, Jinger Pan

**Affiliations:** ^1^ Department of Psychology The Education University of Hong Kong

**Keywords:** Chinese, Word class, Reading, Parafovea

## Abstract

The present study aimed to investigate the parafoveal processing of word class in Chinese reading, focusing on how grammatical category consistency affects word recognition. The gaze‐contingent boundary paradigm was adopted in an eye‐tracking study. In each sentence, a parafoveal target word was replaced with three types of previews: identical to the target word, consistent in word class, or inconsistent in word class. The nonidentical previews were designed to be neither semantically nor contextually related to the target words or the surrounding sentence context. The findings revealed that consistent word class previews significantly facilitated the processing of target words when they were subsequently fixated on, compared to inconsistent word class previews, resulting in a word class preview benefit. This effect was observed across both early and late eye movement indices, regardless of the target word's grammatical category. Our findings provide compelling evidence for the early processing of word class information in the parafovea, independently of semantics or word class predictability, highlighting a word class preview benefit. These findings emphasize the early integration of syntactic properties in Chinese, offering insights into character‐based language reading.

## 1 Introduction

While reading, readers move their eyes to scan the text and extract information to comprehend the text. They not only process the visual and linguistic properties of the foveal word that is being fixated on, but also the parafoveal upcoming words. Researchers are interested in what kind of information about the parafoveal words could be processed before being fixated on. While most studies investigated the processing of lexical properties such as phonology, orthography, and semantics (Hohenstein & Kliegl, 2014; Pollatsek, Lesch, Morris, & Rayner, 1992; Schotter, 2013; Yan, Richter, Shu, & Kliegl, 2009), a few studies in alphabetic languages have looked at whether syntactic fit of the parafoveal preview word could affect the processing of the target words (Angele & Rayner, 2013; Brothers & Traxler, 2016; Veldre & Andrews, 2018). Studies in Chinese have shown that parafoveal processing of syntactic information was only observed in skipping rates of high‐frequency one‐character target words when comparing  identical  with syntactically infelicitous previews (Zang, Du, Bai, Yan, & Liversedge, 2020). In addition, most of these studies used materials in which the word classes of the target words were highly predictable (over 90%). The current study provided novel evidence for a more general parafoveal preview effect of word class in Chinese.

The boundary paradigm is commonly used to measure parafoveal processing (Rayner, 1975). In this paradigm, there is an invisible boundary between the pretarget word and the target word. Before the readers’ eyes cross this invisible boundary, a preview word is shown at the target word position. When the reader's eyes cross the boundary, the preview word will be immediately replaced by the correct target word. Researchers manipulate the overlap of specific properties (e.g., orthographic, phonological, or semantic features) between preview and target words to examine whether these properties can be processed in the parafovea. If a related preview shortens the fixation duration on the target word when it is later fixated on, as compared to an unrelated preview, it can be concluded that the related preview facilitates the processing of the target word. This facilitation is termed the *preview benefit* (PB).

Over the past decades, the orthographic and phonological PBs have been documented in alphabetic languages (Balota, Pollatsek, & Rayner, 1985; Pollatsek et al., 1992) as well as in logographic scripts, such as Chinese (Liu, Inhoff, Ye, & Wu, 2002; Tsai, Lee, Tzeng, Hung, & Yen, 2004). Early access to higher‐level linguistic properties, such as semantics and morphology of the parafoveal words, has also been reported in Chinese (Pan, Wang, McBride, Cho, & Yan, 2023; Tsai, Kliegl, & Yan, 2012; Yan et al., 2009). This is presumably because the characters in Chinese represent meaning, leading to direct access to semantics from orthography, bypassing phonological processing (Hoosain, 1991; Yan & Kliegl, 2023), resulting in a semantic PB. Early semantic PBs have also been observed in languages with highly regular orthography‐phonology correspondence, such as German (Hohenstein & Kliegl, 2014) and Korean (Yan, Wang, Song, & Kliegl, 2019). This is likely due to the fast access to phonological representations from the visual words, allowing time to access semantics (Hohenstein, Laubrock, & Kliegl, 2010). However, the semantic PB in English has only been observed when there is a strong semantic association between the preview and the target words (Schotter, 2013). Recent evidence has established that semantic preview effects in English depend on the acceptability of the preview word as a continuation of the sentence (e.g., Veldre & Andrews, 2016).

As for parafoveal syntactic processing, Angele, Laishley, Rayner, and Liversedge (2014) found that for three‐letter English words, word frequency instead of syntactic fit of the word affects the decision to skip or to fixate on that word. Several later studies have shown that word class could be processed in the parafovea as manifested in both skipping and fixation duration measures (Brothers & Traxler, 2016; Snell, Meeter, & Grainger, 2017). However, their results could not distinguish the influence of word class from contextual plausibility (Veldre & Andrews, 2016, 2017). Veldre and Andrews (2018) further demonstrated word class PBs in two experiments. When the preview words were contextually implausible in the target sentence (e.g., she eventually found a spare *stool* behind the crowded bar), a preview that is consistent with the target in word class (*uncle*) facilitated the processing of the target word (*stool*) as compared to the inconsistent word class preview (*begin*) only in gaze durations (GDs, the cumulative fixation durations of all fixations in first‐pass reading) and in second‐pass reading times. Experiment 2 manipulated more subtle syntactic cues (subject‐verb agreement and verb tense) for words within the same word class. Readers fixated for a shorter period on the target word (e.g., *refuel*) in the target setence (e.g., *Her plane will probably refuel later than expected this afternoon*.) when the preview was semantically and syntactically plausible (e.g., *depart*) compared to when the preview was semantically plausible but syntactically invalid (e.g., *landed*) in first‐fixation durations (FFDs, duration of the first‐fixation on a word in first‐pass reading, irrespective of the number of fixations a word receives) as well as in GDs and in second‐pass reading times. These suggested that very early extraction (as reflected in FFD) of word class information in the parafovea relies on contextual plausibility. Similarly, in Korean (Kim, Radach, & Vorstius, 2012), incorrect case markers prolonged fixation duration on the pretarget word as well as GD and total reading time (TRT, sum of durations of all fixations on a word in first‐ and second‐pass reading) on the target word. These case markers are suffixes that are attached to Korean nouns to indicate their roles in the sentence (e.g., subject, object). While they do not carry lexical meaning like noun stems, they serve an important syntactic function. Therefore, Kim et al. provided critical evidence that syntactic information from parafoveal words can be encoded.

Although parafoveal syntactic processing in natural sentence reading has not been thoroughly studied in Chinese, its theoretical importance should not be overlooked because the writing system and syntactic structure differ fundamentally from English. Unlike English, Chinese relies more on word order and function words than on inflectional morphology, meaning that syntactic cues are distributed differently. In fact, Chinese provides relatively few syntactic cues compared to languages that use subtle syntactic information, such as subject‐verb agreement, verb tense, and case marking. Therefore, investigating whether parafoveal syntactic processing occurs in Chinese will clarify whether such effects are universal or shaped by orthographic and structural properties. Yang, Wang, Chen, and Rayner (2009) adopted the violation paradigm and studied the processing of the syntax of foveal words during Chinese sentence reading. They replaced a critical word in the sentence with words that are either of the same word class or of a different word class. By comparing these two conditions, they found a word class effect in the target region in GD and TRT. The influence of word class also spilled over to the region after the target region, suggesting early access of syntax in Chinese reading.

As for parafoveal processing of word class, Zang, Du, Bai, Yan, and Liversedge (2020) compared the identical preview and the word class inconsistent preview in Chinese. They found that word class inconsistent preview disrupted the processing of the target word. However, the identical preview and the word class inconsistent preview not only differed in terms of grammatical category in relation to the target word, but also in word frequency (i.e., a high‐frequency identical preview was compared with a low‐frequency word class inconsistent preview, and vice versa), as well as their orthographical, phonological, and semantic relations to the target word. Thus, a pure word class PB could not be concluded. Lu, Fu, Zhang, Zang, and Bai (2022) tested parafoveal processing of word class in single‐character words but did not find differences between the consistent and inconsistent previews in skipping or fixation duration measures. Recently, Lu (2025) tested the effect using two‐character verb‐object phrases. A parafoveal target phrase (e.g., “弹琴” [play the piano]) was paired with an identical, a syntactically consistent (e.g., “弹窝” [play the nest], verb‐noun but semantically implausible), and a syntactically inconsistent (e.g., “弹答” [play the answer], verb‐verb violating both syntax and semantics). With high word class predictability (96% anticipating a noun after the verb in the target phrase), the results showed a selective syntactic preview effect limited to reduced skipping probability (SP; the probability of a word not being fixated on during first‐pass reading) in the inconsistent condition compared to the consistent condition, with no differences in FFD, single fixation duration (SFD; the duration of the fixation on a word when the word receives only one fixation during the first‐pass reading), or GD, suggesting early syntactic processing.

It should be noted that there was a high constraint on the word class of the target words in these studies across languages (over 90% of participants predicted a word class consistent with the target word). It calls into question whether it is the word class itself or the prediction of the word class that leads to the facilitation of processing the target word. Moreover, Lu et al. (2022) tested single‐character high‐frequency words, which were highly likely to be skipped (over 60%). The nonidentical two‐character phrases did not form a real word in Lu (2025), and the exact noun in the target phrase was quite predictable (over 50%). It is not clear whether the same findings could be generalized to a nonpredictable situation among two‐character real words, which constitute about 70% of Chinese words. The present study aimed to investigate the pure parafoveal processing of word class when the target words are with a low word class constraint and the previews are contextually related.

Nouns and verbs differ in semantics, phonology, syntax, and other linguistic levels. Evidence from neuropsychology, electrophysiology, and neuroimaging studies has consistently shown that words of different grammatical categories are processed differently in various writing systems (e.g., Barber, Kousta, Otten, & Vigliocco, 2010; Kellenbach, Wijers, Hovius, Mulder, & Mulder, 2002; Paliti, Ben‐Shachar, Hendler, & Hadar, 2007; Shapiro & Caramazza, 2003; Shapiro et al., 2005; Tsai et al., 2009). How these differences affect behaviors in word retrieval and recognition remains an area of debate. It has been shown that in picture naming tasks, verb retrieval is generally slower and less accurate than nouns. Object‐noun and action‐verb retrieval is shown to be affected by factors such as word frequency and imageability (see Abel, Maguire, Naqvi, & Kim, 2015, for a review). In a picture naming task in Chinese, Lu et al. (2001) also found that disyllabic verbs were named more slowly than nouns. However, such a difference was not significant in an auditory shadowing task. Furthermore, when nouns and verbs were presented in isolation, some studies failed to find differences in neural correlates between the two classes of words (Chan et al., 2008; Li, Jin, & Tan, 2004; Yang, Tan, & Li, 2011). Thus, whether nouns and verbs are recognized or retrieved differently may depend on the tasks and measures. Although many of the abovementioned studies offer high spatial resolution for precisely localizing brain regions involved in syntactic processing, they lack the temporal resolution needed to capture the time course of word‐class processing. The time course of noun and verb recognition during sentence reading also needs to be further examined. To address these limitations, eye‐tracking is a vital technique for examining early, parafoveal processing of word classes.

The present study aimed to investigate parafoveal word class processing and whether such an effect is modulated by the word class of the target word. Given the early word class processing in the fovea during Chinese reading (Yang et al., 2009), we hypothesize that Chinese readers can extract word class information from the parafovea early on, even without the support of contextual plausibility. That is, when the preview word is semantically unrelated to the target word or the sentence context. For the influence of word class on the time course, if verbs impose greater processing difficulty as in picture naming (see Abel et al., 2015, for a review), we expect that when the target word is a noun and the preview word is a verb, the preview may impose greater cost for the processing of the noun target word, leading to a greater word class PB than when the preview is a noun and the target is a verb. However, if verbs and nouns share a similar time course in processing as shown in Lu et al. (2001), we expect a similar word class PB for nouns and verbs.

## 2 Method

### 2.1 Participants

Based on previous studies of a similar kind (e.g., Veldre & Andrews, 2018; Zang et al., 2020) and considering the duration of the experiment, we aimed to collect valid data from 60 participants, with each condition having about 20−25 items. Seventy participants^1^ (*M*_age_ = 21.4 years, *SD* = 3.2 years, 58 females) took part in the eye‐tracking experiment. To prepare the experimental materials, three rating tests were conducted, namely, a semantic relatedness rating test, a cloze predictability test, and a plausibility rating test, with 30 participants in each test. All participants were native Chinese‐speaking university students with normal or corrected‐to‐normal vision. All experimental protocols were reviewed and approved by the Human Research Ethics Committee of The Education University of Hong Kong, and all participants provided written informed consent to have their data collected and published.

### 2.2 Design and material

We adopted a 2 (target word class, TWC) × 3 (preview type) within‐subject design. There were two levels of TWC, that is, noun and verb. It adopted a between‐item design. Preview type had three levels ‐ identical, consistent, and inconsistent word class ‐ and was manipulated within items.

We created 144 sets of two‐character simplified Chinese materials, each consisting of an identical, a consistent word class, and an inconsistent word class preview. For noun target words, the inconsistent word class previews were always verbs. For verb target words, the inconsistent word class previews were nouns. Word class information was taken from SUBTLEX‐CH (Cai & Brysbaert, 2010). We only selected words that are used exclusively as nouns or verbs in our materials. Table 1 shows an example for each TWC. The word frequency and number of strokes of the preview words across the two TWC conditions were matched (*F*s < 1, *p*s > .05). The consistent and inconsistent word class previews were equally semantically unrelated to the target words, and the difference between the TWC was not significant (*F*s < 1, *p*s > .05).

**Table 1:** Word properties

Target word		Preview type		
		Identical	Consistent word class	Inconsistent word class
Noun		商人	丛林	相比
	Meaning	businessman	forest	compare
	Word class	noun	noun	verb
	Log frequency	2.62 (0.41)	2.59 (0.37)	2.63 (0.41)
	No. of strokes	15.46 (1.58)	15.44 (1.60)	15.36 (1.72)
	Semantic relatedness	NA	1.79 (0.45)	1.74 (0.45)
	Predictability (%)	3.5 (9.6)	0.0 (0.0)	0.0 (0.0)
	Plausibility	4.16 (0.40)	2.76 (0.81)	2.72 (0.70)

Verb		宣布	引起	专家
	Meaning	announce	induce	expert
	Word class	verb	verb	noun
	Log frequency	2.57 (0.39)	2.57 (0.41)	2.57 (0.43)
	No. of strokes	15.40 (1.70)	15.42 (1.69)	15.35 (1.72)
	Sematic relatedness	NA	1.84 (0.37)	1.74 (0.52)
	Predictability (%)	2.3 (6.2)	0.0 (0.0)	0.0 (0.0)
	Plausibility	4.11 (0.40)	2.80 (0.74)	2.77 (0.72)

*Note*. An example set of critical words. The meanings, word class categories, means of log‐transformed word frequency (occurrences per million) based on Cai and Brysbaert (2010), stroke counts, semantic relatedness ratings on a 5‐point scale, predictability, and plausibility ratings on a 5‐point scale.

**Fig. 1:**
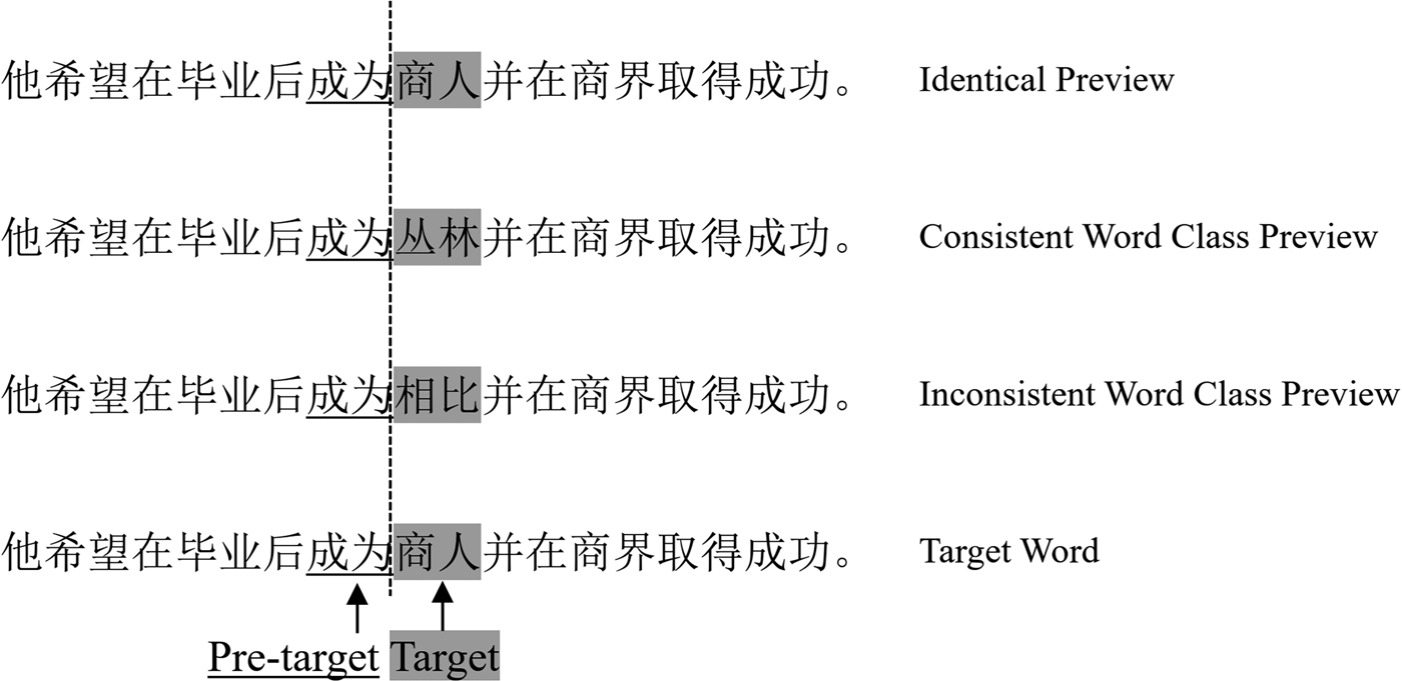
A set of an example sentence with a noun as the target word, which is parafoveally primed by different conditions of previews. To better illustrate the experimental procedure, the preview words and the target words are highlighted. The pretarget words were underlined. However, they were presented normally during the experiment. An invisible boundary was placed between the pretarget and target words (as indexed by the vertical line). When the reader's gaze crosses this boundary, the preview word is immediately replaced by the target word. The target sentence translates as: *He wishes to become a businessman and excels in the business industry upon graduation*.

Fig. 1 shows an example sentence with different preview conditions. The pretarget words were all two‐character words. The context of the experimental sentences was neutral, as reflected by the cloze predictability test. Participants were given the first half of the sentences up to and including the pretarget words and were instructed to complete the sentences. Each participant completed 72 sentences. The preview words in the two nonidentical conditions (i.e., consistent and inconsistent word class previews) were not correctly guessed in the cloze test. For identical previews, nouns were correctly guessed 38 times out of 1080 guesses (3.5%), and verbs were guessed 25 times correctly out of 1080 observations (2.3%). The target words were guessed more often than the nonidentical previews (*F* (1,142) = 18.614, *p* < .001). But there was no difference between target verbs and nouns, and their interaction with preview types was not significant (*F*s <1, *p* > .10). In the plausibility test, participants were given the first half of the sentences up to the preview/target word position, and they were asked to rate how likely the sentences were to be continued and completed as meaningful sentences. Similar to the predictability test, the identical previews were more plausible than the two nonidentical previews (*F* (1,142) = 268.735, *p* < .001). However, the difference between the consistent and inconsistent word class previews was not significant across the two TWC conditions (*F*s <1, *p* > .10). In order to investigate a more general word class preview effect, we did not specifically design sentences with high constraints on the target word class. To validate this, we analyzed the participants' responses in the cloze test (Veldre & Andrews, 2018). For noun target sentences, 57% (*SD* = 25%) of the inputs in the target word positions were nouns, and for verb target sentences, 56% (*SD* = 31%) of the inputs were verbs. There was no significant difference between noun and verb target sentences (*t* = 0.267, *p* > .05). Both predicted word class percentages were significantly lower than in previous studies (Lu et al., 2022; Veldre & Andrews, 2018; Yang et al., 2009).

### 2.3 Apparatus

The EyeLink 1000 Desktop system (SR Research) with a sample rate of 1000 Hz was used to record participants’ eye movements. Sentences were displayed in a single horizontal line on a 24‐inch ASUS VG248QE monitor (resolution: 1920×1080 pixels; refresh rate: 144 Hz). The Song font was used, and each character (48 pixels) subtended an approximate visual angle of 1.0°. The participants were seated 70 cm from the monitor, and were tested individually with their heads secured on a forehead‐and‐chin rest. Recordings and calibrations were conducted monocularly, focusing on the right eye, while participants viewed the content binocularly.

### 2.4 Procedure

Prior to the experiment, participants’ gaze positions were calibrated using a 5‐point grid, with a maximum error margin of less than 0.5°. Tracking accuracy was verified before the presentation of each sentence. The initiation of the subsequent sentence was triggered by the participant's gaze on the initial fixation point, aligning the first character of the sentence with this point. In the event that the eye‐tracker did not detect the gaze around the fixation point, an additional calibration was conducted. Participants were instructed to read the sentences silently for comprehension, then to fixate on a designated point in the lower‐right corner of the monitor, and subsequently press a keyboard button to indicate the completion of a trial. Before attempting the experimental sentences, each participant was asked to complete 12 practice trials. To keep participants engaged, a random selection of 48 experimental sentences was followed by a simple yes‐no comprehension question. On average, participants correctly answered 87.9% (*SD* = 5.9%) of the questions.

### 2.5 Data analysis

Fixations were determined utilizing the algorithm for detecting saccades in Engbert and Kliegl (2003). Several steps were taken to screen the data. Initially, a total of 502 trials (5.5%) were deleted due to tracker errors, participants’ blinks, or body movements. Target words with FFDs shorter than 60 ms or longer than 800 ms, as well as GDs exceeding 1000 ms, were also excluded (*n* = 75, 1.1%). Furthermore, trials with regressions originating from the pretarget or target words (*n* = 461, 5.4%) were not analyzed, due to their potential indication of incomplete lexical processing (Briihl & Inhoff, 1995). Finally, trials involving early display changes (i.e., saccades that triggered the display change but ultimately landed to the left of the boundary, also known as “j‐hooks”; *n* = 386, 4.5%) and late display changes (i.e., display changes triggered during a post‐boundary fixation; *n* = 341, 4.0%) were also removed. There were 5643 observations in total for fixation duration analyses. They were evenly distributed across the experimental conditions.

Linear mixed models (LMMs) and general LMMs were used to analyze continuous fixation duration measures, that is, FFD, GD, and TRT, and categorical SP. Data were analyzed using the lme4 package (Version 1.1‐34; Bates et al., 2015a) within the R programming environment (R Core Team, 2023). By analyzing different fixation measures, we inferred the time course of the word class processing. Effects appearing in FFD are typically attributed to an earlier phase of processing compared to those observed only in GD, which involves refixations on the target word. Effects exclusively detected in second‐pass reading indicators, like TRT, indicate a later processing stage (Inhoff, 1984; Inhoff & Radach, 1998).

We employed a sum contrast for the TWC and an orthogonal Helmert‐contrast for the preview type. The first level of the Helmert contrast compared the inconsistent and consistent previews, revealing a word class preview effect. The second level compared the identical preview with the mean of the two nonidentical previews, revealing an identical PB. Participant‐ and item‐related variance components were specified as random effects. Parsimonious LMMs for successful convergence (Bates, Maechler, Bolker, & Walker, 2015b; Matuschek, Kliegl, Vasishth, Baayen, & Bates, 2017) were reported. The final models included random intercepts for items and participants, as well as participant‐related random slopes for the identical PB and for the target‐word class. *P*‐values were calculated using the lmerTest package (Version 3.1‐2; Kuznetsova, Brockhoff, & Christensen, 2017). Dependent variables for viewing times were log‐transformed in the LMMs (Kliegl, Masson, & Richter, 2010). Data and scripts for data analysis are available at https://osf.io/8gwrt.

## 3 Results

Table 2 shows the means and SDs of SP, FFD, GD, and TRT. Identical previews were skipped more often than nonidentical previews (see Table 3). The result may not provide strong evidence for a clear plausibility effect on SP. Although the identical previews were more plausible than the two nonidentical previews, they were also predicted more frequently. Therefore, the observed effect might be attributable to predictability, plausibility, or a combination of both. On average, over all three preview conditions, verb target words were skipped more than noun target words. All other effects were not significant.

**Table 2:** Eye movement measures

	Preview type			
Target word		Identical	Consistent word class	Inconsistent word class
Noun	SP	23.5 (17.9)	19.5 (16.8)	22.6 (18.8)
	FFD	243 (35)	293 (69)	307 (69)
	GD	262 (46)	333 (96)	341 (89)
	TRT	267 (50)	349 (105)	353 (93)
Verb	SP	25.9 (18.1)	24.8 (19.2)	24.2 (17.6)
	FFD	243 (36)	287 (69)	298 (66)
	GD	261 (50)	320 (95)	343 (100)
	TRT	266 (52)	336 (98)	354 (105)

*Note*. Means and standard deviations (in parentheses) for skipping probability (SP) in percent, first‐fixation duration (FFD), gaze duration (GD), and total reading time (TRT) in ms. Values were computed across participant means.

**Table 3:** Model outputs

	Skipping probability				First‐fixation duration				Gaze duration				Total reading time			
Fixed effect	Est.	SE	*z*‐value	*p*‐value	Est.	SE	*t*‐value	*p*‐value	Est.	SE	*t*‐value	*p*‐value	Est.	SE	*t*‐value	*p*‐value
Intercept	−1.475	0.144	−10.249	**< .001**	5.559	0.022	247.451	**< .001**	5.639	0.027	205.094	**< .001**	5.668	0.028	199.714	**< .001**
WC.PB	−0.047	0.034	−1.376	.169	−0.018	0.005	−3.427	<.001**	−0.022	0.006	−3.688	**< .001**	−0.016	0.006	−2.582	**.010**
ID.PB	0.046	0.020	2.309	**.021**	−0.055	0.005	−10.769	< .001**	−0.069	0.006	−11.083	**< .001**	−0.077	0.006	−12.326	**< .001**
TWC	0.216	0.068	3.160	**.002**	−0.013	0.011	−1.212	.229	−0.017	0.014	−1.208	.230	−0.018	0.014	−1.250	.215
WC.PB × TWC	0.129	0.069	1.877	.061	0.006	0.010	0.628	.530	−0.013	0.012	−1.033	.302	−0.013	0.012	−1.026	.305
ID.PB × TWC	−0.024	0.039	−0.634	.526	0.009	0.006	1.569	.117	0.008	0.007	1.200	.230	0.008	0.007	1.228	.220

Abbreviations: ID.PB, the identical preview versus the mean of the two nonidentical previews; TWC, target word class; WC.PB, the consistent word class preview versus the inconsistent word class preview.

As shown in Table 3, in all three fixation measures, the main effects of TWC were not significant. Most importantly, the word class PBs were significant for FFD, GD, and TRT. The readers fixated on the target words for a significantly shorter amount of time when the preview words were of the same word class as the target words, as compared to when they were of a different word class. The interactions between these word class PBs and TWC were not significant, suggesting that the word class PBs applied to both types of target words. The identical PBs were significant across all fixation measures. The interactions between TWC and the identical PBs were not significant.

## 4 Discussion

The present study examined the parafoveal processing of word class information. Results showed that readers fixated for shorter durations on the target word when the preview was consistent with the target word in terms of word class, compared to when the preview was of a different word class. Such a word class PB was observed in FFD, and it persisted to GD and TRT. However, we did not observe this effect in SP. An exploratory analysis was carried out that included syntactic predictability based on the cloze test (i.e., whether the predicted word shares the word class with the target word) as a predictor. The interaction between word class preview and word class predictability was not significant in any of the fixation measures (*p*s > .05). Our results suggested that parafoveal word class processing does not rely on the prediction of the word class contextual plausibility. Furthermore, word class processing is initiated early on, before a word is fixated.

In alphabetic languages, the parafoveal plausibility effects observed in previous studies were the combined effects of word class and contextual plausibility of the preview (Brothers & Traxler, 2016; Veldre & Andrews, 2016, 2017; Snell et al., 2017). Veldre and Andrews (2018) differentiated the influence of these two factors in two experiments. The time course of this effect depends on the contextual plausibility. When the preview words were contextually plausible, the effect of word class was observed in skipping as well as in FFD, and it persisted into second‐pass reading times. However, when preview words were contextually implausible, the word class PB was observed in GD and second‐pass reading times. In our study, the preview words in the consistent and inconsistent word class conditions were neither semantically related to the target word nor contextually plausible. In agreement with Veldre and Andrews (2018), we did not find a word class preview effect in SP. It is, therefore, not clear if a combined effect of semantics and syntax would elicit an effect in determining whether a word would be skipped or fixated.

Different from Veldre and Andrews (2018), we did not include previews that were semantically related to the target words, as we wanted to test the word class effect mainly when there is no semantic overlap. Their word class effect was not observed in FFD when the previews were semantically implausible, while we have shown the word class PB in FFD independent of semantic processing and word class predictability. Although in English, lexical categories and other syntactic information of some words are indicated by explicit syntactic markers (e.g., *‐tion* and *‐ment* as noun markers), in Veldre and Andrews (2018), many of the items did not use words with these markers. In such a case, as the prediction of word class is high (over 90%), participants may rely more on word meaning, as indicated by the early effect of word class when the previews were semantically related to the target words. Chinese lacks such markers to indicate word class. Thus, the sentence context plays an important role in determining the semantic and syntactic properties of words. It was, therefore, proposed that higher‐level processing in Chinese would not be initiated immediately (Aaronson & Ferres, 1986) and that syntactic and semantic processing cannot be dissociated in Chinese (see Zhang, 1997a, 1997b), because semantic information is necessary to determine the syntactic fit of a word in the sentence. Similarly, Yang et al. (2009) combined syntactic violation with semantic violation, in which these two conditions were matched in semantic violation at the whole‐sentence level. Creating a purely syntactic violation without accompanying semantic changes can be extremely challenging, as replacing a noun with a verb (or vice versa) alters not only the syntactic function but also inevitably introduces semantic anomaly, due to the absence of explicit morphological markers in Chinese. However, the difference between the semantic violation condition and the combined semantic‐syntactic violation condition in Yang et al. (2009) suggests, at least to some extent, that words’ syntactic properties can be dissociated from semantics and can be processed early on (see also Wang et al., 2008, using the same paradigm in a functional imaging study). After all, it is fair to conclude that the observed differences between the semantic violation and the double (semantic + syntactic) violation in the critical word (target character) and in the target region (the target character together with the immediate preceding verb) in Yang et al. (2009) may reflect immediate syntactic processing difficulty arising in the context of semantic integration failure. In our study, using the gaze‐contingent boundary paradigm (Rayner, 1975), the preview words would be replaced by the target words once participants’ eyes crossed the invisible boundary. Thus, there would be no semantic violation in the two nonidentical conditions after the display change. As for predisplay change, the plausibility rating shows that the two nonidentical conditions were equally (im‐)plausible. This suggests that the semantic violations of the two nonidentical conditions were matched. Therefore, the effects observed for these two conditions could be attributed to the word class difference. It is possible that, because of the uncertainty of the semantic and syntactic properties of words, in order to comprehend the sentence, Chinese readers give priority to integrating the syntactic properties of words when reading. Specifically, in our current study, when the sentences do not have high constraints on the target word class (below 60%), having fast access to the word class facilitates the comprehension of the sentence structure. Such processing could be different from previous studies in which the word class predictability is extremely high. With low word class predictability, the source of such an effect may not be the violation of syntactic structure beyond the word level, such as phrase or sentence. The processing of word class might be initiated in the bottom‐up direction, as participants did not have a specific expectation of the grammatical category of the upcoming word. Early word class processing, as reflected in SP in a high word class constraint situation, has also been demonstrated in Lu (2025), using verb‐noun two‐character phrases as target words and manipulating the word class of the second character. However, given the low word class constraint in our study, we did not observe the effect in skipping but in early as well as late fixation duration measures among two‐character words. In addition, unlike our study, where two‐character real words were used as previews, the verb‐noun phrases in the nonidentical conditions in Lu (2025) did not form any meaningful units. It is possible that participants segmented the verb‐noun phrases in the nonidentical conditions as two separate units in the parafovea. This could also lead to differences in observing the effects in different measures.

Using single‐character words, Lu et al. (2022) did not find parafoveal word class PB. It should be noted that while they used high‐frequency one‐character target words, our study employed two‐character words as both pretarget and target words. Angele and colleagues (2013, 2014) found that for short words in English, frequency had a more important role in determining whether a word would be skipped or fixated. Adopting the same logic, it is likely that high‐frequency short words used in Lu et al. (2022) eliminated the chance to observe the word class effect. In contrast, a longer processing window and greater informational complexity of disyllabic words may give readers more time and richer cues for linguistic analysis, enabling higher‐level computations such as assigning syntactic roles or anticipating structural relationships.

The initiation of the processing of syntactic properties might depend on the paradigm used. Chen (1999) used the violation paradigm but presented characters one by one to participants in self‐paced reading. By comparing the word class consistent and inconsistent conditions, it was concluded that word class processing was delayed to the post‐target region. In an ERP (event‐related potential) study, Yu and Zhang (2008) also reported difference in the N400 between the combined syntatic category and semantic violations and the correct setences. However, adopting the eye‐tracking technique in natural sentence reading, Yang et al. (2009) demonstrated the word class effect in the target region as well as in the post‐target region. The effects were shown in both first‐pass and second‐pass reading times. Our findings also suggested early access to word class in the parafovea, which persisted to a later stage of processing. Thus, in a natural sentence reading task where words are presented simultaneously, being able to preview the upcoming word may lead to earlier processing of word class. Together, these differences suggest that eye‐tracking is a sensitive tool to study natural sentence processing.

Finally, it was shown previously that nouns and verbs were processed differently. However, in our current study, we did not find evidence that the word class PB differs between the target word classes. On the one hand, it agrees with previous findings in word recognition in terms of time course (Lu et al., 2001). We did not include other word classes. Future studies could further investigate if words of other grammatical categories are processed differently and if contextual constraints regarding part of speech can influence word class processing.

In sum, the current study provides further evidence for pure parafoveal word class processing in low contextual plausibility and low word class predictability sentences, demonstrating that syntactic properties could be processed early on.

## Author contributions

Zijun Qi and Yue Xi contributed equally to this study.

## Conflict of interest

The authors declare no conflict of interest.

## Note

1 Data from seven participants in the eye movement experiment were excluded due to low comprehension accuracy (< 80%). The eye movement data reported here were based on the remaining 63 participants.
